# Carbon dots: synthesis, sensing mechanisms, and potential applications as promising materials for glucose sensors

**DOI:** 10.1039/d4na00763h

**Published:** 2024-11-22

**Authors:** Kawan F. Kayani, Dlzar Ghafoor, Sewara J. Mohammed, Omer B. A. Shatery

**Affiliations:** a Department of Chemistry, College of Science, Charmo University Peshawa Street, Chamchamal Sulaimani City 46023 Iraq; b Department of Chemistry, College of Science, University of Sulaimani Qliasan St Sulaimani City Kurdistan Region 46002 Iraq kawan.nasralddin@univsul.edu.iq; c College of Science, Department of Medical Laboratory Sciences, Komar University of Science and Technology Sulaymaniyah 46001 Iraq; d Department of Anesthesia, College of Health Sciences, Cihan University Sulaimaniya Sulaymaniyah City Kurdistan Iraq; e Research and Development Center, University of Sulaimani, Kurdistan Regional Government Qlyasan Street Sulaymaniyah 46001 Iraq

## Abstract

The disruption of glucose (Glu) metabolism in the human body can lead to conditions such as diabetes and hyperglycemia. Therefore, accurately determining Glu levels is crucial for clinical diagnosis and other applications. Carbon dots (CDs) are a novel category of carbon nanomaterials that exhibit outstanding optical properties, excellent biocompatibility, high water solubility, low production costs, and straightforward synthesis. Recently, researchers have developed various carbon dot sensors for fast and real-time Glu monitoring. In this context, we provide a comprehensive introduction to Glu and CDs for the first time. We categorize the synthetic methods for CDs and the sensing mechanisms, further classifying the applications of carbon dot probes into single-probe sensing, ratiometric sensing, and visual detection. Finally, we discuss the future development needs for CD-based Glu sensors. This review aims to offer insights into advancing Glu sensors and modern medical treatments.

## Abstract 2

The study on the synthesis of carbon dots, sensing mechanisms, conditions associated with glucose imbalance, and potential applications as promising materials for glucose sensors.

## Graphical abstract

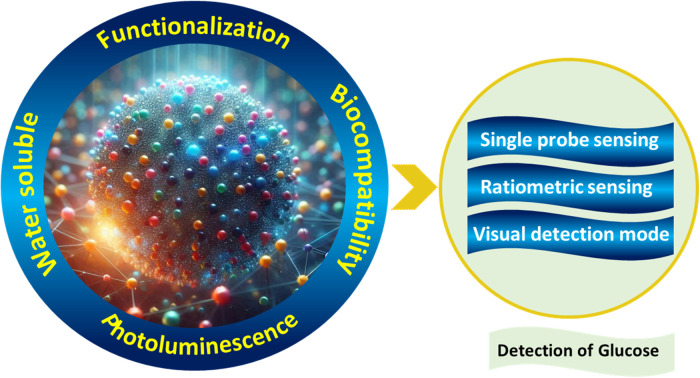

## 1. Introduction

Diabetes mellitus is a condition resulting from a defect in insulin secretion, leading to the buildup of Glu. In the year 2000, over 170 million people globally were affected by diabetes mellitus, and it is projected that by 2030, this number will increase to 366 million.^1^ Glu levels in the human body are crucial indicators of overall health. For individuals with diabetes, it is especially important to monitor these levels on a daily basis.^2,3^ As a result, there is a pressing need to develop highly sensitive, selective, and reliable methods for Glu detection that can be applied across various fields, including clinical diagnostics, biotechnology, and the food industry.^4,5^ To date, a variety of methods and devices have been proposed for Glu testing, including electrochemical sensors,^6–9^ Raman spectroscopy,^10,11^ chemiluminescence (CL),^12,13^ electrochemical transistor sensors,^14,15^ potentiometric sensors,^16,17^ and fluorescence (FL) sensors.^18–22^ Among these, FL sensors have gained recognition as a simple, rapid, and convenient tool for Glu detection.

Various fluorescent materials have been utilized for the detection of Glu, including metal–organic frameworks (MOFs),^23–26^ nanoclusters (NCs),^27^ and small molecule fluorescent sensors.^28,29^ However, each of these materials has certain limitations. MOFs struggle with poor solubility, nanoclusters have issues with short-term stability, and small molecule-based sensors suffer from poor selectivity.^30^ In recent years, CDs have attracted considerable interest across various fields.^31–37^

As innovative 0-dimensional materials with diameters ranging from 1 to 10 nm, CDs exhibit excellent photostability, good solubility in water, and resistance to photobleaching.^38–40^ Compared to other fluorescent materials, CDs offer advantages such as low cost, ease of use, mild preparation conditions, low toxicity, biocompatibility, and a wide range of sources.^40–42^ Consequently, they have been extensively utilized in optoelectronics,^34,43,44^ catalysis,^33,45^ biomedical applications,^46,47^ industrial applications,^32^ food studies,^48^ and particularly in analytical chemistry as fluorescent probes.

Additionally, for clinical applications, the optical and surface chemical properties of CDs make them ideal candidates for theranostic uses, including biosensing,^49^ bioimaging^50^ and drug delivery,^51^ both *in vitro* and *in vivo*. In diagnostics, fluorescence molecular bioimaging is crucial for early tumor detection, allowing noninvasive, highly sensitive, and specific observation of pathological and physiological processes.^52^ CDs offer several advantages, such as low cytotoxicity, excellent biocompatibility, stable photoluminescence, easy functionalization, and remarkable resistance to photobleaching.^43,53^ Due to these benefits, numerous *in vivo* and *in vitro* bioimaging applications have been developed recently.^54–56^ Moreover, CDs are widely utilized in next-generation optical biosensors due to their high sensitivity, low cost, and practicality. These sensors are used not only for elemental and biomarker detection but also for monitoring changes in fluorescence signals at the single-cell level, through mechanisms such as fluorescence enhancement (turn-on) or quenching (off-state).^57,58^

CDs were first developed in 2004.^59^ Since then, their simple synthesis and unique properties have led to a substantial increase in publications exploring various precursors and synthesis methods. These methods are generally divided into top-down and bottom-up approaches.

CDs can be synthesized using two primary methods: “top-down” and “bottom-up.” Top-down techniques, such as ultrasonic synthesis and chemical exfoliation, break down larger carbon structures and are suited for large-scale production but often require extended processing times, harsh conditions, and expensive equipment.^31,60^ On the other hand, “bottom-up” methods, which are typically used for producing CDs, involve constructing CDs from molecular precursors such as Glu and citric acid through processes such as microwave pyrolysis and chemical vapor deposition. These methods provide better control and produce CDs with fewer defects.^61,62^

**Fig. 1:**
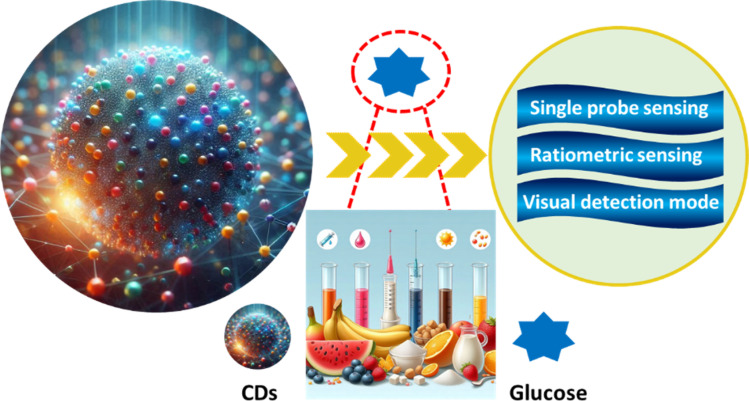
Summary of CDs and their applications.

In this review, we aim to offer a unique perspective by highlighting recent progress in using CDs for detecting Glu in different samples. We will cover the synthesis of CDs and examine practical applications for Glu detection, including single probe sensing, ratiometric sensing, and visual detection methods in real samples. Additionally, we will briefly address the challenges and future prospects in the development and application of CDs, as illustrated in Fig. 1.

## 2. Conditions associated with glucose imbalance

In recent decades, various factors such as shifts in eating habits and physical activity have contributed to an increase in disorders linked to abnormal blood Glu levels. Alongside new discoveries regarding diseases associated with this issue, these developments have driven research to explore innovative methods for Glu monitoring.^63,64^ The condition linked to abnormal blood Glu levels is known as Diabetes Mellitus (DM), which manifests in several forms: type 1 DM, type 2 DM, Alzheimer's disease, cardiovascular disease and kidney disease.

Type 2 DM is the most prevalent form, accounting for around 90% of all diabetes cases, and the number of patients continues to increase.^65^ An estimated 374 million people are at increased risk of developing this form of the disease. Type 1 diabetes is more common among children and adolescents, with over 1.1 million young individuals living with it. The two most common types of diabetes stem from either the pancreas's inability to produce insulin (DM1) or reduced tissue sensitivity to insulin (2 DM), both leading to disrupted carbohydrate metabolism. Therefore, monitoring blood Glu levels is crucial.^66,67^

Similar to Alzheimer's disease (AD), type 2 diabetes mellitus (2 DM) is another widespread condition often linked to obesity and aging. In the U.S., approximately 24 million people exhibit clinical symptoms of 2 DM.^68,69^ This condition is characterized by elevated blood Glu levels due to increased hepatic Glu production, reduced insulin production by pancreatic β-cells, and insulin resistance, where target cells have a diminished response to insulin as a result of down-regulated expression of insulin receptors (IRs), IGF-1 receptors (IGF-1R), and insulin receptor substrate (IRS) proteins.^70^ Several clinical studies have found a connection between 2 DM and neurodegenerative disorders, including memory decline. Longitudinal studies have shown that Glu intolerance and impaired insulin secretion are associated with a higher risk of developing dementia or AD.^70–72^

Diabetes mellitus, being a condition caused by either insufficient insulin or insulin resistance, falls under the field of endocrinology. Compared to non-diabetic individuals, people with diabetes face a significantly higher risk of experiencing cardiovascular events, along with worse outcomes from cardiovascular disease, including a notably higher mortality rate.^73^ The American Heart Association even refers to diabetes as “a cardiovascular disease”.^74^ This dual view of diabetes as both an endocrine and systemic vascular disorder is supported by two key factors: (1) the heightened cardiovascular risk related to even early, mild cases of diabetes, and (2) the importance of not only managing blood sugar levels but also identifying and addressing other cardiovascular risk factors to reduce morbidity and mortality in affected patients.^75^

**Fig. 2:**
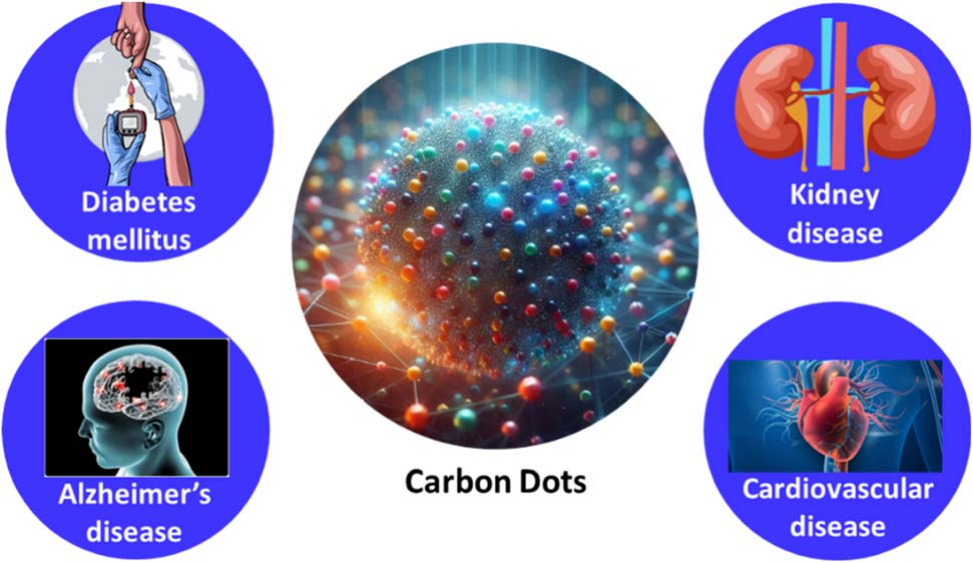
Common glucose-related diseases.

Diabetic kidney disease (DKD) is a leading cause of both morbidity and mortality in individuals with diabetes mellitus and the primary cause of end-stage renal disease globally.^76^ The kidney's critical role in Glu homeostasis is now widely understood. For instance, renal gluconeogenesis significantly contributes to overall Glu production in the postabsorptive state. The kidney helps regulate Glu levels by filtering and reabsorbing Glu. Typically, Glu filtered by the glomeruli is fully reabsorbed, but in cases of hyperglycemia or reduced reabsorptive function, glucosuria can occur,^77^ as illustrated in Fig. 2.

Most current articles on Glu monitoring primarily concentrate on the medical applications of Glu measurement using various sensors. In contrast, there is a scarcity of comprehensive reviews that systematically explore the underlying principles of Glu detection. This review is the first to focus specifically on CDs for Glu detection, opening new avenues for a deeper understanding of this technology and its potential applications.

## 3. Synthesis methods

Compared to other nanoparticles, CDs offer advantages in their synthesis methods, with multiple options available some of which are environmentally friendly^30,78^. They can be produced through a “bottom-up” approach^79,80^ such as using small organic molecules such as glucose^81^ and urea,^82^ or even biological materials such as biowaste,^60^ animal products,^83,84^ and plant extracts,^49^ which chemically assemble into nanoparticles.^85,86^ Alternatively, a “top-down” method involves breaking down larger pure carbon materials such as carbon black,^87^ graphite oxide,^88^ carbon nanotubes,^89^ or graphite into nanoparticles.^90,91^

**Fig. 3:**
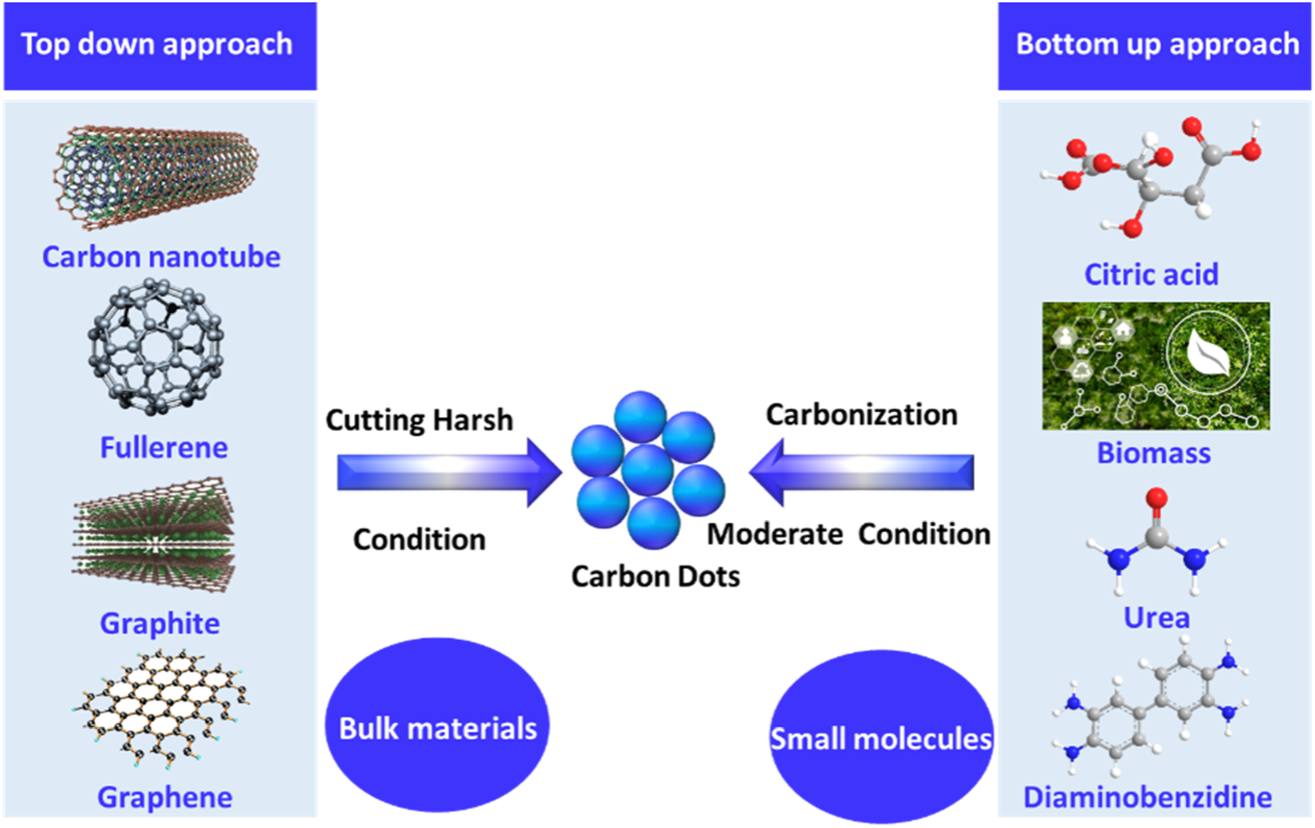
Methods for synthesizing CDs: top-down *versus* bottom-up approaches.

The bottom-up method offers several important benefits over the top-down approach,^92^ including being more eco-friendly^93–95^ and less time-consuming^96^ and enabling simple adjustments to the surface state and composition of the CDs.^97–99^ These benefits make bottom-up methods more prevalent in the literature and contribute to CDs being an ideal choice over other nanoparticle types. In this process, the organic compound is dissolved in a solvent and heated until it experiences dehydration and carbonization.^100–102^ This method can be accomplished using different techniques, including hydrothermal carbonization,^103,104^ microwaving,^105,106^ combustion,^107–109^ and pyrolysis.^110^ Each technique has its own pros and cons regarding efficiency, time, cost, and energy consumption, and they produce CDs of different sizes and compositions,^111,112^ as summarized in Fig. 3.

### 3.1 Bottom up synthesis methods

The bottom-up approach involves hydrothermal carbonization, solvothermal synthesis, microwaving, ultrasonic synthesis, pyrolysis and combustion.

#### 3.1.1 Hydrothermal and solvothermal synthesis of CDs

Hydrothermal carbonization is widely used in scientific research and is considered relatively simple and low-cost.^113,114^ It utilizes non-toxic starting materials to achieve controlled CD formation, enabling straightforward adjustments to their composition.^115,116^ This method requires a specially designed reaction vessel capable of withstanding the high temperatures necessary for carbonization while trapping emitted vapors to raise the reaction pressure.^117–119^ Containing the vapors enhances the efficiency of digesting organic materials, and the sample can be heated longer without significant evaporation loss.^120^ CDs produced through this method typically exhibit high photoluminescence quantum yields;^121^ however, drawbacks include non-uniform particle sizes, impurities that are difficult to remove, and possible variations in photoluminescence behavior among CDs within the same sample.^122,123^ In addition to hydrothermal fabrication, the solvothermal method for preparing CDs offers the benefits of being cost-effective and requiring simple equipment.^124^ Unlike the hydrothermal process, where water is used as the solvent, the solvothermal approach uses one or more solvents in a sealed Teflon-lined steel autoclave.^125,126^ The mixture of solvent and carbon source undergoes a reaction under high pressure and temperature conditions.^127^

#### 3.1.2 Microwave-assisted synthesis of CDs

Microwaves, a form of electromagnetic radiation, can serve as an alternative heating method to ovens used in hydrothermal carbonization. Similar vessels can be employed but made from nonmetallic materials to enhance the digestion efficiency of organic materials.^128,129^ The main benefit of microwaving is the strong interaction between the carbon source and electromagnetic radiation, which allows for fast and localized heating.^130–132^ This approach is energy-efficient, eco-friendly, and generally regarded as simpler than many other CD synthesis methods.^53,133^ Nevertheless, the resulting CDs may have a wide size distribution, and separating them from the solution can be challenging.^134,135^ As a result, CDs are frequently synthesized using microwave-assisted techniques.

#### 3.1.3 Ultrasonic synthesis of CDs

Ultrasonic treatment has been recognized as an effective method for producing various CDs, leading to numerous studies on its application for their synthesis. In this process, carbon precursors, along with acids, alkalis, and other oxidizing agents, are subjected to intense ultrasound waves, causing the carbon particles to break down into nanoscale particles. Continuous cavitation of the molecules occurs during the process. The high energy of the ultrasonic waves eliminates the need for complex post-treatment procedures, enabling the simple synthesis of small-sized CDs.^136,137^ As a result, the ultrasonic synthesis method triggers the reaction using the thermal effects of cavitation and ultra-high-frequency vibrations. It offers unmatched benefits, including environmental friendliness, cost-effectiveness, strong penetration, and consistent results.^138^ Therefore, high-performance CDs can be produced by combining ultrasound with other techniques.

#### 3.1.4 Pyrolysis and combustion synthesis of CDs

Both pyrolysis and combustion involve thermal decomposition methods but differ in atmospheric conditions: pyrolysis occurs in a low-oxygen or an oxygen-free environment, while combustion requires oxygen.^107,110,139,140^ These techniques may require strong acids or bases to initiate the digestion of the carbon precursor, making them less environmentally friendly.^60,141^ CDs synthesized *via* pyrolysis tend to have relatively high photoluminescence QYs, but the process is time-consuming, requires specific setups, and the CDs are difficult to separate from the solution.^142,143^ Additionally, producing CDs through combustion is regarded as one of the most efficient, low-cost, simple, and rapid one-step methods, relying on the carbonization of precursor molecules. Carbonization is a chemical process where organic materials undergo extended pyrolysis in an inert atmosphere, resulting in solid residues with a higher carbon content.^96^

#### 3.1.5 Microfluidic synthesis

The concept of microfluidics was first introduced by Manz in the 1990s.^144^ With advancements in micromachining, microfluidic technology has rapidly evolved, making significant strides in various fields. Compared to traditional chemical synthesis methods, microfluidic technology offers several distinct advantages.^145^ Microfluidic systems, known for their precise control over reaction conditions, have attracted the attention of researchers for generating nanomaterials. Previously, studies on quantum dots (QDs) were mostly conducted in batch reactors. Although these reactors were relatively simple, they struggled with uncontrollable reactions and challenges in maintaining consistency across different batches. In contrast, microfluidic-based reactors provide precise control over reaction conditions, such as temperature, pressure, and concentration distribution.^146–149^ Most bottom-up synthesis methods for QDs involve two key phases: nucleation and growth. These processes can be finely tuned in microreactors to produce QDs with specific sizes, morphologies, and compositions. Furthermore, due to the high sealing efficiency of microreactors, the need for inert gas protection typically required in batch production can often be eliminated in the microfluidic synthesis of many QD types.^150^ Microfluidic approaches to CDs generally enhance synthesis efficiency, increase quantum yields, and have the potential for large-scale production.

### 3.2 Top-down method

The top-down approach encompasses techniques such as arc discharge, laser ablation, electrochemical methods, and chemical oxidation.

The arc discharge method involves continuous electrical discharge between the cathode and anode, which generates high temperatures, gradually consuming the anode and forming CDs.^151^ Since 2004, it has gained popularity as a quick preparation method. However, CDs produced this way have inconsistent particle sizes and uncontrollable morphology, leading to the method being rarely used today.^152,153^ The laser ablation method employs a high-energy laser as a light source to irradiate carbon materials and produce CDs.^154^ This method allowed for the specific functionalization of CD surfaces, enhancing their fluorescence properties.^155^ The chemical oxidation method uses strong oxidizing agents to synthesize CDs. The primary advantage of this method is its ability to enhance the water solubility of CDs, and it remains a widely used technique for this purpose.^156^ The electrochemical method uses graphite rods as the carbon source to produce CDs through electrochemical treatment.^157^ In 2012, Shinde and Dhanraj discovered that this method allows control over the size of CDs by adjusting factors such as potential, electrolyte concentration, and reaction temperature. This method is popular for its simplicity, high yield, and production of uniformly sized particles.^158^

## 4. Sensing mechanism

In theory, any fluorescence variation such as changes in intensity, anisotropy, wavelength, or lifetime associated with the concentration of various analytes can serve as a basis for sensors. This section provides a concise overview of these sensing mechanisms.

### 4.1 Förster resonance energy transfer

**Fig. 4:**
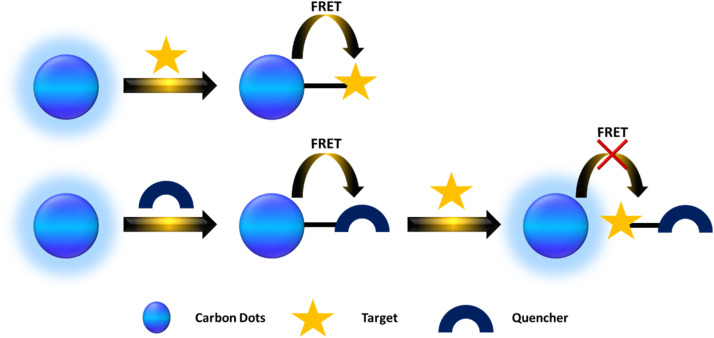
A scheme describing FRET from the CDs to the quencher and target.

FRET (Förster Resonance Energy Transfer) involves the nonradiative transfer of energy between an excited donor and an acceptor through long-range dipole–dipole interactions. In 1948, Theodor Förster introduced an equation that quantifies the efficiency of electronic excitation transfer between an energy donor (D) and acceptor (A).^159^ CDs are frequently utilized as donors. Currently, there are primarily two types of FRET-based CD probes. The first type involves the significant overlap between the emission spectrum of CDs and the absorption spectrum of the target, allowing FRET to occur from CDs to targets when they are at an appropriate distance. This interaction leads to a decrease in the fluorescence intensity of the CDs or a shift in the emission wavelength toward either the blue or red end of the spectrum. In the second scenario, FRET occurs from CDs to the quencher, resulting in reduced fluorescence intensity of the CDs. However, when a target is present, the distance between the CDs and the quencher increases, halting FRET and thereby increasing the fluorescence intensity of the CDs. As illustrated in Fig. 4.

### 4.2 Inner filter effect (IFE)

The fluorescence intensity observed is proportional to the intensity of the excitation light, and the quantum yield is slightly lower than that seen in an infinitely dilute solution. This phenomenon is known as the IFE, which can result in a reduction of the excitation intensity at the observation point or a decrease in the observed fluorescence intensity due to absorption.^160^

**Fig. 5:**
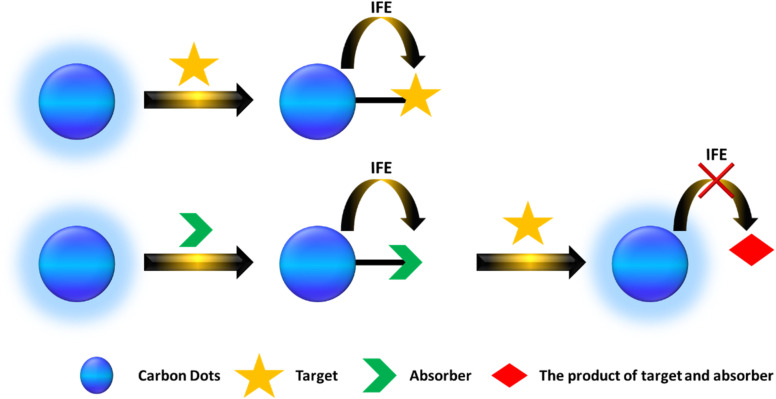
A scheme showing the IFE from the CDs to the quencher and target.

Currently, there are two primary types of IFE-based fluorescent probes. The first type occurs when the absorbance spectrum of the analyte significantly overlaps with the excitation or emission spectrum of CDs, thereby directly inducing the IFE. The second type involves a situation where the absorption spectrum does not align well with the CD spectrum; instead, a component (A) is introduced to react with the analyte to form a product. As illustrated in Fig. 5.

### 4.3 Photoinduced electron transfer (PET)

**Fig. 6:**
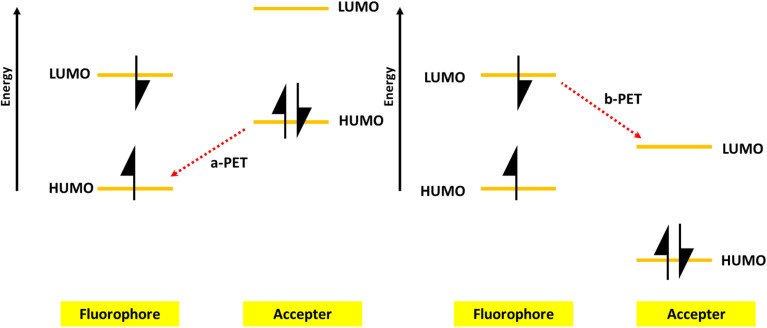
A scheme showing PET from the CDs to the quencher and target.

PET is a process involving the transfer of electrons between an electron donor (D) and an electron acceptor (A). As illustrated in Fig. 6, there are two forms of PET. When excited using light, the electrons in a fluorophore move from the highest occupied molecular orbital (HOMO) to the lowest unoccupied molecular orbital (LUMO). If an acceptor is present with a HOMO energy higher than that of the fluorophore, electron transfer can occur from the acceptor to the fluorophore, a phenomenon known as a-PET. The change in fluorescence of the fluorophore due to a-PET can serve as a signal for detecting the acceptor or target, as the presence of either reduces a-PET. The second form is called b-PET, in which electron transfer takes place from the fluorophore to the acceptor because the fluorophore's LUMO is higher than that of the acceptor.^161^ A decrease in fluorophore fluorescence indicates the presence of the acceptor, while an increase in fluorescence can signal the detection of a target that binds to the acceptor, preventing it from accepting the excited electron from the fluorophore.

### 4.4 Static quenching mechanism

Static quenching occurs when a nonfluorescent ground-state complex is formed through the interaction between CDs and a quencher. When this complex absorbs light, it quickly returns to the ground state without emitting a photon.^162^ Static quenching exhibits several characteristics: (1) the absorption spectrum may change due to the formation of a ground-state complex. (2) The fluorescence may increase with temperature because of the dissociation of the weakly bound quencher. (3) The fluorescence lifetimes of the CDs remain constant. Because the static quenching mechanism can be easily confirmed, it is frequently utilized for analyte detection.

**Fig. 7:**
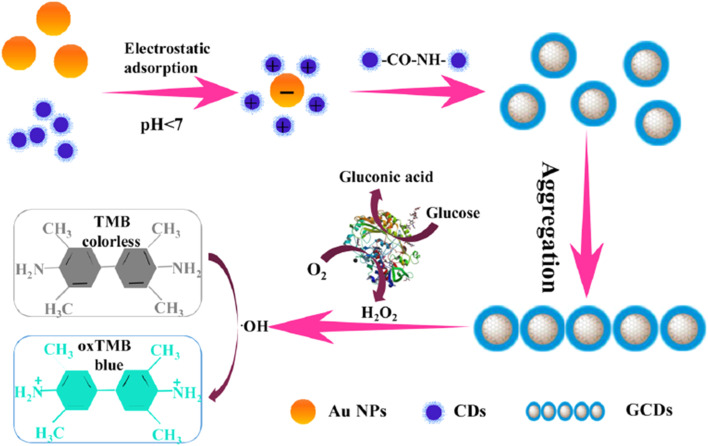
A schematic illustration for the synthesis of GCDs and detection of Glu with permission. Copyright 2021, Elsevier.^167^

## 5. Applications

CDs, owing to their remarkable properties such as exceptional photoluminescence, low toxicity, abundant precursor sources, easy surface functionalization, and excellent biocompatibility, are promising zero-dimensional carbon-based nanomaterials.^163,164^ They are widely used in bioimaging to visualize cells and tissues, providing a non-toxic alternative for diagnosing diseases. CDs also show great potential in drug delivery systems, where they can be functionalized to target specific tissues, enhancing treatment precision. In biosensing, CDs assist in detecting biomolecules such as glucose and cancer markers, enabling early and accurate diagnoses. Additionally, CDs play a role in cancer therapy through photodynamic and photothermal treatments, helping to target and destroy cancer cells. These unique characteristics make them suitable candidates for use as Glu sensors. This review aims to examine cutting-edge CD-based optical sensors and their sensing capabilities.

### 5.1 Single probe sensing

In this section, we explore the use of single-probe sensors based on CDs for precise Glu measurement in various samples. These sensors are not only cost-effective but also exhibit low toxicity, making them ideal for practical applications. The sensor system typically consists of CDs or a combination of CDs with other materials, designed to accurately detect and quantify Glu concentrations. This approach leverages the unique properties of CDs, such as strong photoluminescence and ease of surface modification, to enhance sensitivity and reliability in Glu sensing.

Chen *et al.* developed^165^ a sensitive and selective Glu sensor using thiol-functionalized CDs (S-CDs). Upon exposure to H_2_O_2_, the S-CDs were converted into non-luminous assemblies due to the target-initiated oxidation of –SH groups into –S–S– bonds. Since Glu can be broken down into H_2_O_2_ by Glu oxidase, the S-CDs were used to detect Glu with a low detection limit (LOD) of 0.03 μM, a dynamic range of 0.1–50 μM, and high selectivity for distinguishing Glu from other sugars.

In this study, a molecularly imprinted electrochemical sensor (MIECS) was developed for Glu detection using a glassy carbon electrode (GCE) modified with hollow nickel nanospheres (HNiNSs), CDs, and chitosan (CS). Under optimal conditions, the sensor exhibited two wide linear ranges for Glu concentrations: 0.03–10 μM and 20–300 μM, with a LOD of 4.6 nM. The sensor was successfully applied to detect Glu in human serum and fermentation samples.^166^

Gan *et al.* developed^167^ Au/CDs(GCDs) for Glu detection using the surface-enhanced Raman scattering (SERS) method, which exhibited enhanced peroxidase-like activity. Based on this property, GCD composites were further utilized to detect Glu, achieving a LOD as low as 5 × 10^−7^ M, with a working range of 0 to 50 μM, as shown in Fig. 7.

**Fig. 8:**
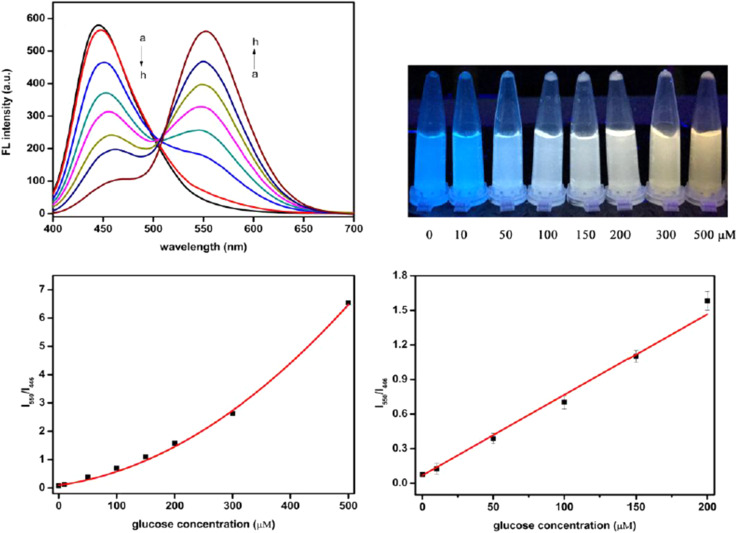
Visual detection of Glu based on CDs with permission. Copyright 2024, Elsevier.^204^

Mutuyimana *et al.* developed^168^ orange-red emissive CDs whose FL is significantly quenched by hydrogen peroxide. Since the oxidation of Glu by Glu oxidase (GOx) generates H_2_O_2_, which quenches the FL through static quenching, a fluorometric method was designed for Glu detection. Under optimal conditions, the method exhibited a linear response for Glu concentrations ranging from 0.5 to 100 μM, with a LOD of 0.33 μM.

Doping CDs with different metal atoms enhances their photophysical properties due to surface passivation. Metal-doped CDs not only modify the photophysical behavior of the CDs but also give them multifunctional characteristics, even though the exact mechanism behind their photoluminescence remains unclear. For example, the synthesized Cu-doped CDs demonstrated peroxidase-like activity that surpassed that of horseradish peroxidase. As a result, the Cu-CD-based CL sensing method was effectively used for sensitive Glu detection, with a low LOD of 0.32 μM and a linear range of 1–48 μM.^169^

In another study, bifunctional Ag-CDs were synthesized, and a simple, sensitive dual-mode sensing platform utilizing colorimetric and SERS techniques was developed for detecting Glu in body fluids. This method took advantage of the Ag-CDs' strong peroxidase-like and SERS activities. The colorimetric detection of Glu showed a linear range of 50–800 μM with a LOD of 11.30 μM, while the SERS mode exhibited a linear range of 10–800 μM with a LOD of 3.54 μM.^170^

In this section, we examine in greater detail the most commonly used CDs for visual Glu sensing platforms, as summarized in Table 1.

**Table 1:** List of selected CDs, probes, real samples, QY, methods, dynamic ranges, and LOD values

Color	Sample	QY%	Method	Dynamic range	LOD	Ref.
Green CDs	Serum	2.74	FL enhancement	0.2–6 μM	0.12 μM	171
Blue NCDs	Urine	—	Electrochemical	1 × 10^−5^ to 5 × 10^−3^ M	3.8 × 10^−7^	172
Green rGO-PBA	—	—	FL quenching	0.01–0.35 M	—	173
Blue Pd-CDs	Fruit juice	—	Colorimetric	0–100 μM	0.2 μM	174
Blue CD/V_2_O_5_	Serum	—	Colorimetric	0.7–300 μM	0.7 μM	175
Green CDs	Serum	71.7	FL quenching	10–240 μM	0.92 μM	176
Blue CDs	Serum	58.7	FL quenching	0.2–20 mM	0.15 μM	177
CDs	—	—	Colorimetric	0–500 μM	87.3 nM	178
Blue Gd-CDs	Serum	—	FL quenching	0.6–40.0 μM	0.6 μM	179
Blue CDs	Serum	—	FL quenching	1–30 mM	1 mM	180
Blue CDs/MnO2	Serum	21.63	FL enhancement	2–200 μM	0.83 μM	181
CDs	Serum	—	Colorimetric	0.010–0.40 mM	2 μM	182
Blue CDs/AgNPs	Serum	—	FL enhancement	2–100 μM	1.39 μM	183
Blue CDs	Serum	—	FL quenching	0.5–7 mM	0.058 mM	184
SCDs	Saliva	22.63	FL quenching	1–250 μM	0.57 μM	185
Green CDs	Milk	—	FL quenching	10–100 μM	0.686 μM	186
Blue CDs	Glial cells	9.91	FL quenching	0.3–15 μM	0.1 μM	187
CDs	Serum	—	Electrochemical	1–12 mM	0.25 mM	188
Blue CDs	Serum	—	FL quenching	9–900 μM	1.5 μM	189
Blue CDs	Serum	9.06	FL quenching	0–30 mM	—	190
Blue CDs	Urine, serum	—	FL enhancement	20–150 μM	3.9 μM	191
Blue CDs	Serum	—	FL quenching	5–750 μM	0.5 μM	192
CDs	Serum	—	Electrochemical	0.5–40 μM, 50–600 μM	0.09 μM	193

### 5.2 Ratiometric sensor

A single probe sensor can be influenced by external factors such as the intensity of excitation light, environmental interference, and probe concentration. These factors may lead to fluctuations in FL intensity, compromising the accuracy and reliability of measurements.^22^ To address these challenges, ratiometric fluorescent probes have been designed, offering self-calibration through two emission peaks that respond differently to the target analyte. Additionally, ratiometric fluorescent probes allow for visual detection by showing color changes under UV light, enhancing both the convenience and efficiency of the analysis process.^194,195^ Developing an innovative ratiometric sensing method holds great promise and importance for the easy detection of Glu.

Cho *et al.* prepared^196^ a ratiometric FL Glu probe based on CDs, where the signal originated from blue CDs and green rhodamine 6G as the reference signal. A change in probe FL color, from blue to green, was observed as the Glu concentration increased. The probe showed a linear range of 0.1–500 μM with a LOD of 0.04 μM. A stable solid-state probe using a hydrogel film exhibited a similar ratiometric FL color change and sensitivity, with a linear range of 0.5–500 μM and an LOD of 0.08 μM.

In this research, a new Fe-doped CD ratiometric probe was fabricated. This probe consisted of blue doped CDs and 2,3-diaminophenazine (DAP), produced by the oxidation of *o*-phenylenediamine (OPD) by Fe-CDs in the presence of H_2_O_2_, which emitted a yellow signal. The responses can be used to accurately quantify H_2_O_2_ and Glu in the ranges of 0–133 μM and 0–300 μM, with LOD of 0.47 μM and 2.5 μM, respectively.^197^

Wen-Sheng and colleagues developed CDs functionalized with boronic acid. This ratiometric probe, which relies on the boronic acid groups on the CDs' surface, interacts with the *cis*-diol groups of Glu to form a coordination compound, leading to FL quenching of the C-dots caused by their aggregation. Moreover, the aggregation of the C-dots simultaneously enhanced resonance light scattering (RLS) due to the presence of two pairs of *cis*-diol groups. As a result, a hybrid ratiometric chemosensor for Glu was designed. Importantly, the inert surface of the CDs allows this probe to measure Glu over a dynamic range of 10 to 2000 μM, with a LOD as low as 10 μM.^198^

A novel ratiometric FL sensor was developed using carbon quantum dots (CQDs) and *o*-diaminobenzene (ODB). The sensor was used to detect various concentrations of Glu standard solutions (as shown in Fig. 8). As the Glu concentration increased, the FL peak of CQDs at 446 nm gradually decreased, while the peak of oxidized ODB (oxODB) at 550 nm steadily increased. To quantify the relationship between the FL changes of CQDs and oxODB and Glu concentration, a linear fit was applied to the FL ratio of oxODB to CQDs against the Glu concentration. The FL ratio demonstrated a strong linear correlation with Glu concentrations in the range of 10 to 200 μM, with a LOD of 1.15 μM.^199^

**Fig. 9:**
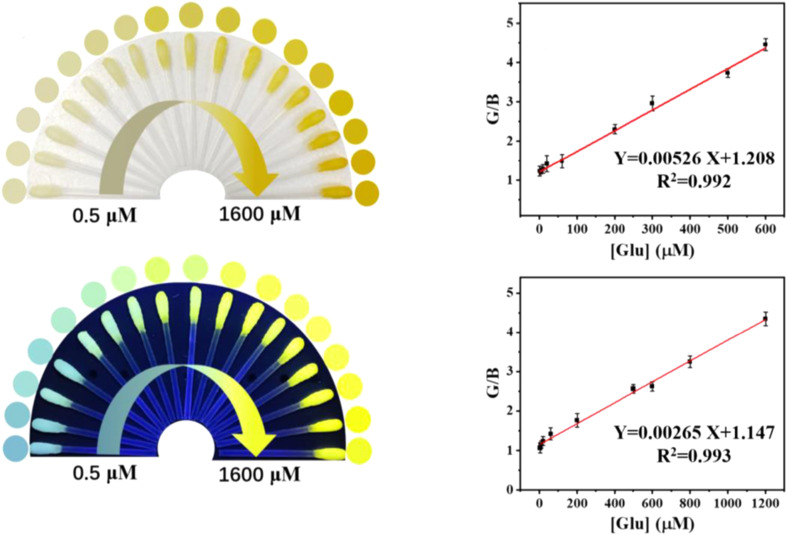
The ratiometric FL spectra for detecting Glu with permission. Copyright 2021, MDPI.^199^

A novel fluorescent probe for Glu detection was developed using a platform combining NCDs and CdTe quantum dots (CdTe QDs). This ratiometric probe, based on blue CDs and yellow CdTe QDs, was successfully applied for Glu detection under optimized experimental conditions. The probe demonstrated a wide linear range of 26–900 μM with a LOD of 7.86 μM.^200^

### 5.3 Visual detection

Colorimetric sensing is a favored approach for analytical chemists, as it enables swift on-site detection through visible color changes.^201^ Visual detection has long captivated scholars, and the development of portable optical sensors for qualitative and semi-quantitative analysis of various substances has further drawn attention from researchers in many disciplines. This method allows analytes to be identified without relying on complex external instruments.^30^ Therefore, creating sensitive and selective methods for visually detecting Glu in various samples is highly important.

In this study, a visual detection method for Glu was developed. Glu was specifically broken down by Glu oxidase (a natural enzyme), generating H_2_O_2_, which was then catalyzed by CDs (FeMn/N-CDs, a nanozyme) to accelerate the conversion of *o*-phenylenediamine (OPD, colorless) into 2,3-diaminophenazine (DAP, yellow). As a result, the absorbance at 450 nm increased with higher Glu concentrations, causing a color change from colorless to yellow. Simultaneously, the FL of FeMn/N-CDs at 450 nm gradually decreased, while the FL of DAP at 550 nm increased, enabling dual-mode detection *via* both ratiometric FL and colorimetry. The portable swabs created for this purpose had dynamic ranges of 1–600 μM (LOD = 0.37 μM) for colorimetric detection and 4–1200 μM (LOD = 1.19 μM) for fluorometric detection,^202^ as shown in Fig. 9.

Bandi *et al.* developed^203^ Fe-doped CDs(FeCDs) with high peroxidase (POD)-like activity, which were immobilized on cellulose nanofibrils (CNFs) to create a composite paper. The POD activity of this paper was assessed through the oxidation of TMB and applied for the colorimetric detection of H_2_O_2_ and Glu. CNFs served as a support for the nanozyme, providing reusability, while also adsorbing the chromogen during the reaction, leading to a visible color change, making it suitable as a test strip for portable, on-site detection. A smartphone was used to monitor the color change, simplifying and reducing the cost of the detection process. This method offered linear detection ranges of 10–70 μM and a LOD of 1.73 μM for Glu.

In this study, N-CDs with excellent peroxidase-like activity were synthesized. These N-CDs catalyzed the oxidation of the chromogenic substrate TMB in the presence of H_2_O_2_, producing a blue oxidized product (TMBox) with an absorption peak at 654 nm. A smartphone-based colorimetric method was developed to quantitatively detect TMBox, recording the 1/*L* values (*L* representing lightness in the HSL color space). Since H_2_O_2_ is a byproduct of Glu oxidation in the presence of Glu oxidase (GOx), this method was adapted for sensitive and selective Glu detection, with a LOD of 1.09 μM.^204^

In this section, we examine in greater detail the most commonly used CDs for visual Glu sensing platforms, as summarized in Table 2.

**Table 2:** List of selected CDs, probes, real samples, QY, methods, dynamic ranges, and LOD values

Color	Sample	QY%	Method	Dynamic range	LOD	Ref.
Green CDs	Human plasma	29.03	Solution assay	1–10 ppm	2.53 ppm (13.98 μM)	205
Blue CDs	Serum and urine	11	Paper platform	10^−6^ to 10^−5^ M	—	206
Blue CDs	Saliva	—	Paper platform	10.0 × 10^−6^, 40.0 × 10^−6^ M	2.60 × 10^−6^ M	207
Green BCNP	Serum	46	Solution assay	32 μM to 2 mM	8 μM	208
Blue Fe-CDs	Serum	19.11	Solution assay	—	—	209
Green R-CDs/B_2_O_3_	Serum	6.70	Portable test gel	0–20 mM	—	210

## 6. Conclusions and prospects

CDs are gaining significant attention due to their exceptional properties, non-toxic nature, availability, and simple synthesis process. Since their discovery, extensive research has been conducted on CDs, with the current focus shifting towards sustainable development, a critical necessity for this century. This review primarily highlights the latest advancements in the use of CDs for the detection of Glu. To advance this field, we have compiled and emphasized reliable synthetic methods for CDs while highlighting key accomplishments in various sensing techniques that utilize CDs for Glu detection. Despite significant progress, several limitations and challenges still require attention.

Despite the progress made in CD research, many challenges remain. Predicting the photophysical behavior and selectivity of CDs for specific targets, such as Glu, is still difficult, as is optimizing CD synthesis. Additionally, inconsistencies in CD properties and selectivity, even when synthesized from the same source, highlight the need for further investigation. The enhanced efficiencies observed in Glu detection also require more in-depth conceptual analysis.

Moreover, the practical use of CDs remains largely confined to laboratory settings. There is an urgent need to advance this technology for real-time, on-site Glu detection and to develop faster, simpler detection methods for broader applications. Extensive research is essential to overcome these challenges and fully realize the potential of CDs in practical scenarios. Additionally, producing CDs in large yields is still of interest and in demand for real industrial applications, making further research on this important aspect necessary.

In conclusion, CD-based fluorescent probes show significant promise for Glu detection, but further research is needed to refine the design of CDs to improve their efficiency in real-world applications. We hope that this review will inspire greater interest in the development and optimization of CDs, ultimately driving their widespread use in Glu detection in the near future.

## Data availability

Data sharing is not applicable to this article as no datasets were generated or analysed during the current study.

## Conflicts of interest

There are no conflicts to declare.
